# Current-induced runaway vibrations in dehydrogenated graphene nanoribbons

**DOI:** 10.3762/bjnano.7.8

**Published:** 2016-01-20

**Authors:** Rasmus Bjerregaard Christensen, Jing-Tao Lü, Per Hedegård, Mads Brandbyge

**Affiliations:** 1 Center for Nanostructured Graphene (CNG), Department of Micro- and Nanotechnology, Technical University of Denmark, Ørsteds Plads, Bldg. 345E, DK-2800 Kongens Lyngby, Denmark; 2 School of Physics, Huazhong University of Science and Technology, 430074 Wuhan, P. R. China; 3 Niels-Bohr Institute and Nano-Science Center, University of Copenhagen, Universitetsparken 5, 2100 Copenhagen Ø, Denmark

**Keywords:** current-induced forces, density functional theory (NEGF-DFT), graphene, molecular electronics

## Abstract

We employ a semi-classical Langevin approach to study current-induced atomic dynamics in a partially dehydrogenated armchair graphene nanoribbon. All parameters are obtained from density functional theory. The dehydrogenated carbon dimers behave as effective impurities, whose motion decouples from the rest of carbon atoms. The electrical current can couple the dimer motion in a coherent fashion. The coupling, which is mediated by nonconservative and pseudo-magnetic current-induced forces, change the atomic dynamics, and thereby show their signature in this simple system. We study the atomic dynamics and current-induced vibrational instabilities using a simplified eigen-mode analysis. Our study illustrates how armchair nanoribbons can serve as a possible testbed for probing the current-induced forces.

## Introduction

The electronic and transport properties of graphene has been the focus of intense study since its discovery in 2004 [1]. Due to the strong σ-bonding between carbon atoms, graphene has a very high thermal conductivity, and can potentially sustain much higher current intensities than other materials. Graphene nanoribbons (GNR) exhibit a bandgap opening due to quantum confinement in the transverse ribbon direction. This opens the possibilities of realizing various electronic devices, especially field-effect transistors, using graphene nanoribbons. Atomically precise ribbons [2], as well as more advanced ribbon-based structures [3–4], have been fabricated “bottom-up” on metal surfaces. The conductance through the ribbons has been investigated using STM [5], and the signals of electron vibrations in the current have been addressed by theory [6].

When cutting graphene into one-dimensional ribbons, dangling bonds emerge at the boundary carbon atoms. If there is an electrical current passing through the ribbon, we expect that these boundary atoms with dangling bonds are mechanically weak compared to the central atoms. Actually, it has been observed experimentally that these atoms can be removed by the passing electrical current due to current-induced local heating [7–8]. One theoretical study suggests that the carbon dimers at the armchair edge vibrate locally and interact strongly with the electrical current [8]. They can be thought as atomic scale defects at the boundary. How the current-induced forces affect the dynamics of these dimers is an interesting question to ask since it could be addressed by experiments. Employing a semi-classical Langevin approach, we have previously studied the current-induced atomic dynamics of a graphene nano-constriction [9]. However, the number of atoms involved even in such a small system makes a simple analysis difficult.

On the other hand, since the first prediction that current-induced forces are nonconservative with respect to energy [10], there has been a substantial theoretical effort aimed at exploring its consequences for the stability of current-carrying nano-systems [11–15], or the possibility of driving atomic motors [10,16]. Moreover, it has been shown that, in addition to the nonconservative force, the current-induced forces also include an effective Lorentz force or pseudo-magnetic force, originated from the Berry phase of electrons [11]. Performing similar analysis using a scattering theory approach shows that the predictions apply equally well to much larger mesoscopic coherent conductors [16–17]. A requirement of impact of the nonconservative force is that two or more vibrational modes close in frequency couple to each other via the current carrying electronic states. This can establish a generalized circular “water-wheel” motion, either in real space [10] or in mode space. Another requirement is that these modes have little damping due to the coupling to the phonon reservoir. Unfortunately, there has not been a clear experimental setup where these new theoretical findings can be put to a test proving their effect in an unambiguous way. Thus, it is of interest to be able to propose such a setup based on first principles calculations with realistic unadjustable parameters.

In this paper, we study the current-induced dynamics in a partially dehydrogenated armchair graphene ribbon. We show that, atomic motion of the dehydrogenated carbon dimer at the nanoribbon boundaries are relatively decoupled from other dimers and also from the rest carbon atoms. This results in several nearly degenerate atomic vibrations, where each of these involves mainly one dimer. However, a coupling of the dimer vibrations takes place via the flowing electrical current. All these features are favorable to observe the effect of current-induced forces, thus making armchair nanoribbon an ideal candidate to study.

In the rest of the paper, we summarize our theoretical (section “Theory”) and numerical (section “Numerical Calculation”) methods, and present our analysis of the armchair graphene ribbon (section “Results and Discussion”). We end this paper with our concluding remarks (section “Conclusion”).

## Theory

**Figure 1:**
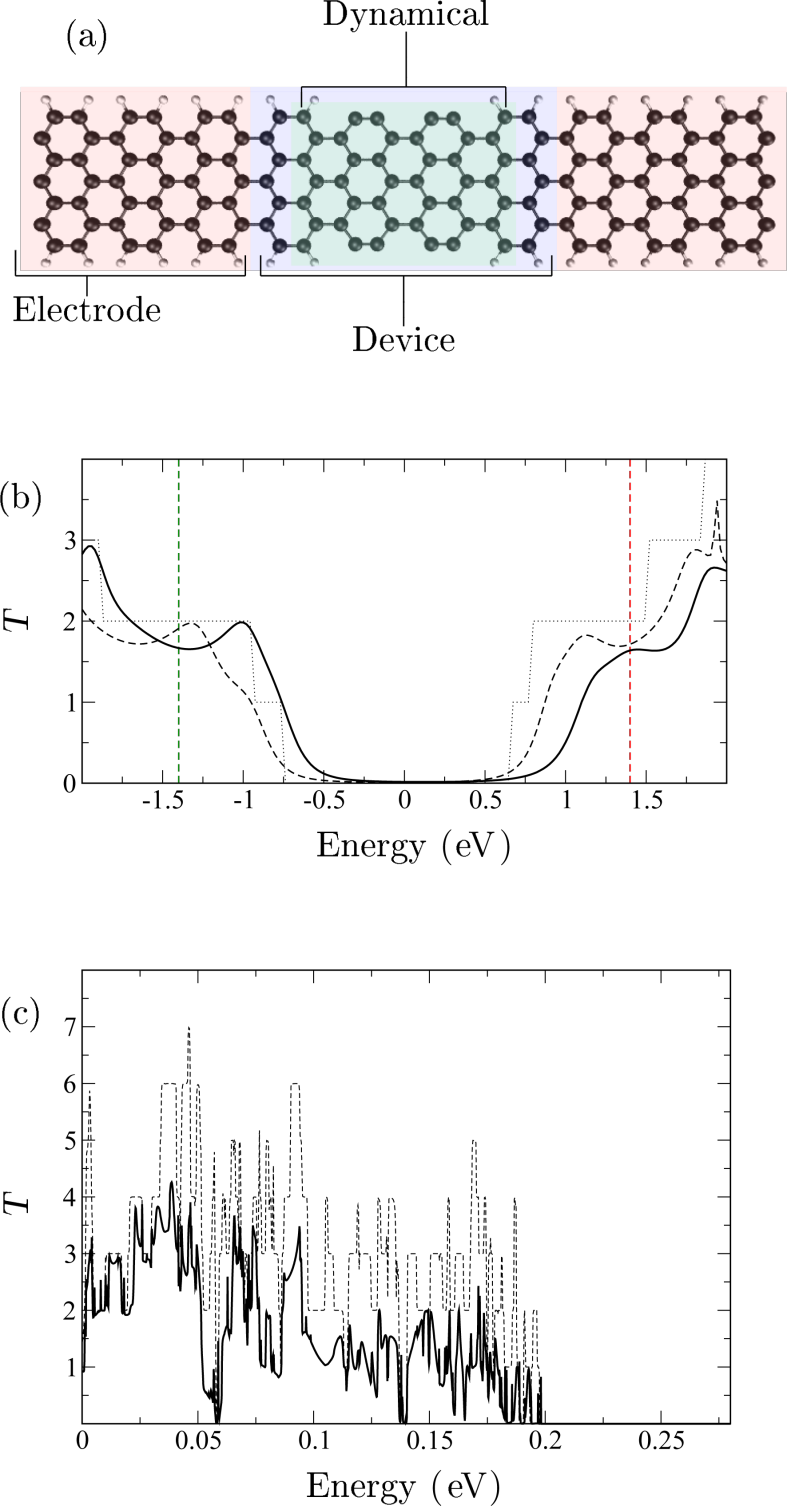
(a) Structure of the transport setup defining device and symmetric electrode(left shown) regions. The motion of atoms is considered in the dynamical region. (b) Electron transmission in a perfect, infinite ribbon (dotted), and with broadened states in the electrodes to mimic metallic contacts with/without dehydrogenation (dashed/full). (Red and green vertical dashed lines indicate the shifts in Fermi energy used below in Figure 2). (c) Solid line is phonon transmission for the structure in (a), dotted line is phonon transmission for a pristine hydrogenated ribbon.

We consider a standard Landauer-type transport setup described in Figure 1a. The system/device is in contact with the left and right leads. Each lead serves as both electronic and phononic bath. The bath degrees of freedom are non-interacting. We are interested in the atomic dynamics in the device region (displacements *U*), which can be described by the semi-classical generalized Langevin equation (SGLE) [12,18–20],

[1]
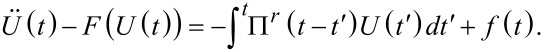


Here *F*(*U*) is the force between atoms in the device region and *f*(*t*) is a random force due to thermal and voltage-bias-induced fluctuations. The Π*^r^* (self-energy) describes the time-delayed back action of the bath on the system due to the motion of the system. It has three contributions,

[2]
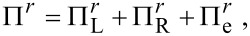


where 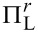 and 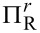 describe the coupling to the phonon reservoirs outside device, while the self-energy 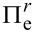 describes the coupling to the electrons. In equilibrium, this result has been obtained by, i.e., Head-Gordon and Tully [21]. In principle we may apply the SGLE including the non-linear part of *F* [22], but here we will restrict ourselves to the harmonic approximation, *F*(*U*) = −**K***U*.

### Non-equilibrium

The semi-classical Langevin equation can be extended to include the non-equilibrium effects in the electronic system due to the current [11–12,23]. In accordance with intuition the “traditional” Joule-heating is present in the fluctuating force, *f*, while the current-induced forces show up in 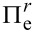. Here we focus on the latter. The contribution from the electron degrees of freedom including the non-equilibrium effects can be expressed in terms of the coupling-weighted electron–hole pair density of states, Λ,

[3]
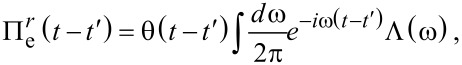


with Λ (including spin), given by,

[4]
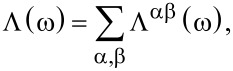


[5]
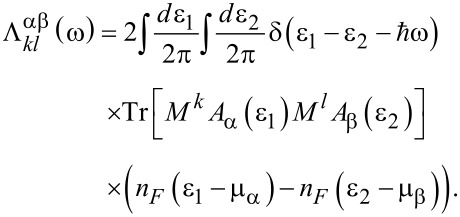


Here, *A*_α/β_ is the density of scattering states incoming from left and right electrodes (indices α and β), while *M* describes the electron–phonon couplings (*k* and *l* phonon indices). One can loosely think of the motion of phonon *k* excites an electron–hole pair of energy 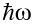 which is absorbed by phonon *l*.

The SGLE, in Equation 1, is given in the time domain. However, since we are considering steady state, it is convenient to work in the frequency domain. Thus, by Fourier transformation we obtain,

[6]
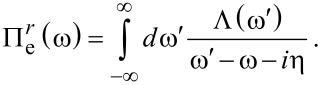


By applying the Sokhatsky–Weierstrass theorem Π*^r^*(ω) can be split into four contributions giving rise to the four forces

[7]
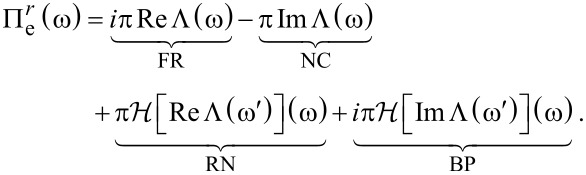


Here, FR, NC, RN, BP represent the electronic friction, nonconservative force, renormalization of the atomic potential, and Berry-phase-induced pseudo-magnetic force, respectively [12].

#### Run-away modes

In order to analyze the influence of the current we define the nonequilibrium phonon density of states (DOS) as,

[8]
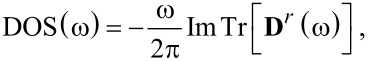


where **D***^r^*(ω) is the nonequilibrium phonon Greens function obtained from the SCLE,

[9]
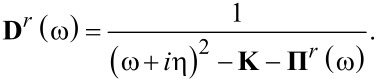


Note that we introduced boldface to underline that these are matrices with mode-index (*k*,*l*). Contrary to the equilibrium situation, the DOS given in Equation 8 can take negative values at certain peak values, due to the electronic current. We can interpret a negative peak in the DOS at a frequency ω_0_ as modes at ω_0_ with a negative lifetime, i.e., with growing in amplitude as a function of time and denote these by ”run-away” modes.

#### Mode analysis

**Figure 2:**
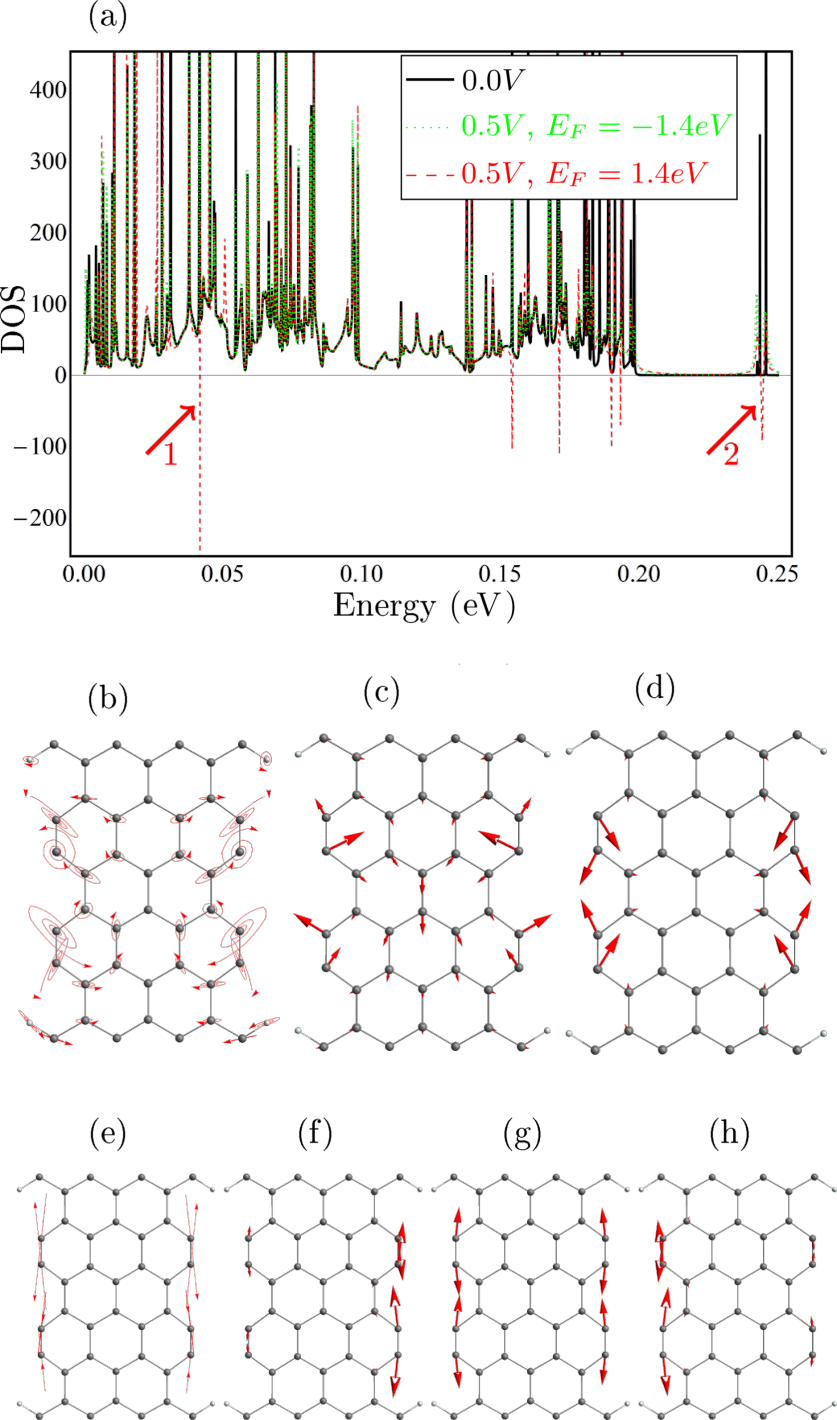
(a) The black solid line shows the phonon density of states excluding the self-energy due to the electronic degrees of freedom. The green dotted and the red dashed lines show the phonon DOS including the current-induced forces for an applied bias of 0.5 V, shifting the Fermi energy to *E*_F_ = −1.4 eV and *E*_F_ = 1.4 eV, respectively, corresponding to the vertical lines in Figure 1b; (b) the run-away mode giving rise to the dip in (a) indicted by arrow 1 (*Q* ≈ 10^−3^); (c,d) the most important normal modes taking part in (b); (e) the run-away mode giving rise to the dip in (a) indicted by arrow 2 (*Q* ≈ 0.5·10^−3^); (f–h) the normal modes taking part in (b).

In order to identify the modes that can show run-away behavior, we need to find the solutions to Equation 1 by setting the driving noise force, *f*(ω), to zero. This is done by treating the velocity and displacement as independent variables and use the relation 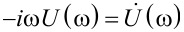 to obtain the double-sized eigenvalue problem. However, the self-energy **Π***^r^* is frequency-dependent. Thus, to analyze a specific runaway mode giving rise to a negative peak in Figure 2a (see below), we evaluate the self-energy at the negative peak frequency ω_0_ as given below in Equation 10,

[10]
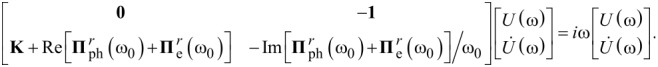


Thus, the dynamical matrix is renormalized by 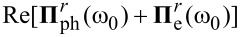 and the friction originates from 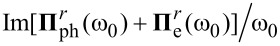. Solving Equation 10 gives a set of eigenmodes and complex eigenfrequencies, but only the “self-consistent” mode, which fulfills Re(ω) = ω_0_, is relevant.

For a given eigenmode a corresponding positive imaginary part of the eigenfrequency designates that the mode is a run-away mode, while if the imaginary part is negative the mode is damped. The damping can be quantified by the inverse *Q*-factor giving the change in energy per period

[11]
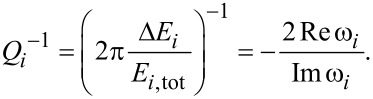


Thus, the run-away modes can be identified as the modes where Im(ω) *>* 0. The run-away modes are a linear combination of the non-perturbed normal modes. Normally, the runaway makes closed loops in real or in abstract mode space. Thus, the NC force allows the mode to pick up energy every time a loop is completed, eventually leading to break down of the harmonic approximation, ending with, e.g., rupture or damping by anharmonic effects leading to a limit cycle motion [24].

## Numerical Calculation

We have calculated the electronic and phononic structure of the graphene nanoribbon from density function theory (DFT) using the SIESTA/TranSIESTA codes [25–26]. The generalized gradient approximation is used for the exchange–correlation functional, and a single-ζ polarized basis set is used for the carbon and hydrogen atoms. A cut-off energy of 400 Ry is used for the real-space grid. The electron-vibrational coupling is calculated using the INELASTICA package, which uses a finite difference scheme [27].

## Results and Discussion

The partially dehydrogenated graphene nanoribbon we considered is shown in Figure 1a, where four hydrogen atoms have been removed on each side of the ribbon. In principle, dehydrogenation could be performed at chosen positions with an STM [28]. The same structure has been considered in our recent work, focusing on the asymmetry in phonon emission and heat distribution due to the nonequilibrium for lower voltage than where run-away instability occurs [29]. This asymmetry is intrinsically linked to momentum transfer from electrons to phonons and thus to the nonconservative current-induced forces (“electron wind”) [30]. As we mentioned before, the reason we choose this structure is that the dehydrogenated carbon dimers can be considered as “defects” in the armchair ribbon. In general defects give rise to modes localized around the defect. They originate from the local change in force constants shifting the mode out of its unperturbed subband [31].

The relative motion of the two carbon atoms in each dimer only couples weakly to the motion of neighbouring dimers and to the phonons in the leads. This high-energy mode is shifted out of the entire phonon bandstructure. Meanwhile, the flowing electrical current passing through these dimers introduce a small bias-dependent coupling via the self-energy (**Π***_e_*) in Equation 10. In principle, one can tune the relative distance between different dimers by changing the ribbon width or the position of the dehydrogenation. It is an ideal and clean system to study the current-induced atomic dynamics, with some tunability since one may imagine doping or gating to shift the Fermi level, *E*_F_, as well as changes in geometry such as varying the distance between dimers.

In this study, the nanoribbon has a width of 7 dimers corresponding to a C–C edge distance of 7.5 Å. The lateral confinement introduces a direct semi-conducting band gap, giving rise to the gap in the electronic transmission for the perfect ribbon as shown in Figure 1b. We have introduced a broadening of the electronic states as in Christensen et al. [6] to mimic coupling to metallic electrodes. This in effect smoothens the transmission curves Figure 1b (dotted lines) akin to the experimental conductance [5]. The introduction of the defects results mainly in a potential shift, but besides this does not impact the transmission dramatically, as seen in Figure 1b (solid lines).

In order to characterize the phonons we show the phononic transmission in Figure 1c. The phonon transmission shows a significant reduction of approx. 50% for 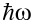 above 25 meV. The fact that we see little difference between a perfect and a defect system at low phonon energy is expected when the wavelength is much larger than the defect. We note that, ideally, the translation (*T*) in the three spatial directions and rotation around the longitudinal direction of the GNR should lead to perfect *T* = 4 at 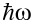, equal exactly to zero for both pristine and defected structure. The deviation is due to our numerical neglect of long-range elastic forces. However, while the low energy/long wavelength modes are important for heat transport, we are here concerned with modes with a higher frequency above 25 meV, where the calculation is expected to be accurate.

The influence of the current-induced forces on the phonon self-energy depend on the underlying electronic properties. Thus, besides the unperturbed phonon DOS exclusive the current-induced forces (black solid line in Figure 2a), we also calculate the non-equilibrium DOS using Equation 8 shifting the Fermi energy away from the gap to *E*_F_ = −1.4 eV (green dotted line) and *E*_F_ = 1.4 eV (red dashed line), and applying a bias of *V*_b_ = 0.5 V. Comparing these results, we see that there are several run-away modes (negative peaks) for *E*_F_ = 1.4 eV, but not for *E*_F_ = −1.4 eV. In Figure 2b and Figure 2e we show two typical run-away modes marked as 1 and 2 in Figure 2a. In Figure 2c,d and Figure 2f–h, we also plot the “bare” normal modes (without coupling to electron and phonon baths), which give the largest contribution to the two selected run-away modes. These run-away modes have in common that they are spatially localized, meaning a vanishing damping due to the coupling to the phonon reservoirs, which is a prerequisite for the run-away instability here. The driving of the motion by the current has to exceed this damping. Both run-away modes involve mainly the dehydrogenated carbon dimers, but while mode 2 lies outside the bulk phonon bands and is thus localized, the frequency of mode 1 is well within the entire phonon band, illustrating that this is not a necessary requirement. In the case of 1 the localization is due to a shift out of a ribbon phonon sub-band. Most of the “bare” normal modes (Figure 2c,d and Figure 2f–h contributing to run-away motion can be considered as a in or out of phase combination of different dimer vibrations.

The two selected modes illustrate how the run-away modes can display circular motion in real-space or in abstract mode space. For the modes showing circular motion it is intuitively clear why these have been dubbed “water-wheel” modes [10], and that the direction of rotation is linked to the direction of current via (angular) momentum transfer [30]. Mode 1 is made up by two principal bare modes, while mode 2 does so in abstract mode space, and consists of mainly three bare modes. In both cases the nonconservative force pump energy into these modes when they oscillate around closed loops. We note that anharmonic coupling will lead to additional damping, which is not included in the harmonic approximation applied here, but we expect a lower anharmonic coupling to the mode well outside the bulk band (mode 2) due to the frequency mismatch. The structure we consider has mirror symmetry in the lateral direction perpendicular to the transport. The resulting motion of the run-away mode respects this symmetry. But, the motion along the current direction is asymmetric since the current breaks the symmetry. This is more obvious for mode 1 (Figure 2c). Mode 2 is strictly localized in the center, but mode 1 has weak coupling to the leads. Consequently, mode 1 shows larger asymmetry. We should mention that this asymmetry in the local heating already shows up before the run-away modes emerge [29].

## Conclusion

In conclusion, we have studied the effect of current-induced forces on the dynamics of dehydrogenated carbon dimers at the edges of graphene armchair ribbon. These carbon dimers are weakly coupled to each other and the rest of the carbon atoms, but they interact with the electrical current. This induces effective coupling between them, and the nonconservative and effective magnetic force become important in describing their dynamics. Using a simplified eigenmode analysis, we analyze how the carbon dimer motion is modified by these forces at different Fermi level positions. The possibility of observing the atomic structure of the two-dimensional structures in microscopy, gating or doping, and atomic scale modification of graphene ribbon boundaries makes it an ideal candidate to study current-induced forces in nanoconductors, where interesting theoretical predictions are awaiting for experimental confirmation.

So far current-induced motion and desorption have been observed around edges in graphene sheets [7–8]. One signature of the nonconservative forces is, besides the asymmetry build into the momentum transfer, the highly non-linear heating of modes with bias [11], which in principle could be observed around edges [32].

## Acknowledgements

We acknowledge computer resources from the DCSC, and support from Center for Nano-structured Graphene (Project DNRF58). J.T.L. acknowledges support from the National Natural Science Foundation of China (Grants No. 11304107 and No. 61371015).

## References

[1] Castro Neto A H, Guinea F, Peres N M R, Novoselov K S, Geim A K (2009). Rev Mod Phys.

[2] Cai J, Ruffieux P, Jaafar R, Bieri M, Braun T, Blankenburg S, Muoth M, Seitsonen A P, Saleh M, Feng X (2010). Nature.

[3] Cai J, Pignedoli C A, Talirz L, Ruffieux P, Söde H, Liang L, Meunier V, Berger R, Li R, Feng X (2014). Nat Nanotechnol.

[4] Liu J, Li B-W, Tan Y-Z, Giannakopoulos A, Sanchez-Sanchez C, Beljonne D, Ruffieux P, Fasel R, Feng X, Müllen K (2015). J Am Chem Soc.

[5] Koch M, Ample F, Joachim C, Grill L (2012). Nat Nanotechnol.

[6] Christensen R B, Frederiksen T, Brandbyge M (2015). Phys Rev B.

[7] Jia X, Hofmann M, Meunier V, Sumpter B G, Campos-Delgado J, Romo-Herrera J M, Son H, Hsieh Y-P, Reina A, Kong J (2009). Science.

[8] Engelund M, Fürst J A, Jauho A P, Brandbyge M (2010). Phys Rev Lett.

[9] Gunst T, Lü J-T, Hedegård P, Brandbyge M (2013). Phys Rev B.

[10] Dundas D, McEniry E J, Todorov T N (2009). Nat Nanotechnol.

[11] Lü J-T, Brandbyge M, Hedegård P (2010). Nano Lett.

[12] Lü J-T, Brandbyge M, Hedegård P, Todorov T N, Dundas D (2012). Phys Rev B.

[13] Dundas D, Cunningham B, Buchanan C, Terasawa A, Paxton A T, Todorov T N (2012). J Phys: Condens Matter.

[14] Cunningham B, Todorov T N, Dundas D (2014). Phys Rev B.

[15] Todorov T N, Dundas D, Lü J-T, Brandbyge M, Hedegård P (2014). Eur J Phys.

[16] Bustos-Marún R, Refael G, von Oppen F (2013). Phys Rev Lett.

[17] Bode N, Kusminskiy S V, Egger R, von Oppen F (2011). Phys Rev Lett.

[18] Feynman R P, Vernon F L (1963). Ann Phys.

[19] Caldeira A O, Leggett A J (1983). Physica A.

[20] Schmid A (1982). J Low Temp Phys.

[21] Head-Gordon M, Tully J C (1995). J Chem Phys.

[22] Lü J-T, Gunst T, Hedegård P, Brandbyge M (2011). Beilstein J Nanotechnol.

[23] Brandbyge M, Hedegård P (1994). Phys Rev Lett.

[24] Bode N, Kusminskiy S V, Egger R, von Oppen F (2012). Beilstein J Nanotechnol.

[25] Soler J M, Artacho E, Gale J D, García A, Junquera J, Ordejón P, Sánchez-Portal D (2002). J Phys: Condens Matter.

[26] Brandbyge M, Mozos J-L, Ordejón P, Taylor J, Stokbro K (2002). Phys Rev B.

[27] Frederiksen T, Paulsson M, Brandbyge M, Jauho A-P (2007). Phys Rev B.

[28] Wang Y F, Kröger J, Berndt R, Vázquez H, Brandbyge M, Paulsson M (2010). Phys Rev Lett.

[29] Lü J-T, Christensen R B, Wang J-S, Hedegård P, Brandbyge M (2015). Phys Rev Lett.

[30] Todorov T N, Dundas D, Paxton A T, Horsfield A P (2011). Beilstein J Nanotechnol.

[31] Savin A V, Kivshar Y S (2013). Phys Rev B.

[32] Islam M S, Tamakawa D, Tanaka S, Makino T, Hashimoto A (2014). Carbon.

